# Complete Genome Sequence of *Paenibacillus polymyxa* YC0573, a Plant Growth–Promoting Rhizobacterium with Antimicrobial Activity

**DOI:** 10.1128/genomeA.01636-16

**Published:** 2017-02-09

**Authors:** Hu Liu, Chengqiang Wang, Yuhuan Li, Kai Liu, Qihui Hou, Wenfeng Xu, Lingchao Fan, Jian Zhao, Jianyu Gou, Binghai Du, Yanqin Ding

**Affiliations:** a College of Life Sciences, Shandong Key Laboratory of Agricultural Microbiology, National Engineering Laboratory for Efficient Utilization of Soil and Fertilizer Resources, Shandong Agricultural University, Taian, China; b College of Resources and Environment, Shandong Agricultural University, Taian, China; c State Key Laboratory of Nutrition Resources Integrated Utilization, Linshu, China; d Zunyi Tobacco Monopoly Administration of Guizhou, Zunyi, China

## Abstract

*Paenibacillus polymyxa* strain YC0573 is a plant growth–promoting rhizobacterium with antimicrobial activity, which was isolated from tobacco rhizosphere. Here, we report the complete genome sequence of *P. polymyxa* YC0573. Antifungal and antibacterial genes were discovered.

## GENOME ANNOUNCEMENT

*Paenibacillus polymyxa* is considered to be a plant growth–promoting rhizobacterium (1, 2). *P. polymyxa* promotes plant growth through different mechanisms, such as biological nitrogen fixation (3), indole-3-acetic acid production (4, 5), enhancement of iron absorption (6), and inducing system resistance of plants (7). *P. polymyxa* can secrete fusaricidin (8), lantibiotic (9), and polymyxin (10) against plant pathogens. *P. polymyxa* strain YC0573 was isolated from tobacco rhizosphere in Guizhou, China, and possesses great potential for inhibiting *Phytophthora parasitica* var. *nicotine*, which causes the black shank of tobacco.

The genomic sequencing of *P. polymyxa* YC0573 was performed using the PacBio platform (11). Up to 104,376 reads totaling 860,491,256 bp were obtained (~140-fold coverage). The largest read contained 42,492 bp. All reads were *de novo* assembled using Canu version 1.3 (12). The genome sequence of *P. polymyxa* YC0573 was annotated by The NCBI Prokaryotic Genome Annotation Pipeline (PGAP) (http://www.ncbi.nlm.nih.gov/genome/annotation_prok). The Carbohydrate-Active EnZYmes database (CAZy) version 20141020 (http://www.cazy.org) (13) was used to analyze the genes encoding carbohydrate enzymes. The gene islands were predicted by IslandPATH-DIMOB (14) and SIGI-HMM (15). The secondary metabolism clusters were predicted by antiSMASH version 3.0.5 (http://antismash.secondarymetabolites.org) (16).

YC0573 contains a chromosome of 6,126,117 bp, with 46.498% G+C content. A total of 5,334 genes were predicted, including 5,017 coding genes, 37 rRNAs, 99 tRNAs, four ncRNAs, and 177 pseudogenes. Four CRISPR arrays were also identified. The 321 genes encoding carbohydrate enzymes were analyzed, including 154 glycoside hydrolases, 61 glycosyl transferases, 13 polysaccharide lyases, 48 carbohydrate esterases, eight auxiliary Activities (AA), and 37 carbohydrate-binding modules; 32 gene islands and three prophages were found throughout the genome. As expected, the genes involving secondary metabolism were identified, such as one gene cluster for polymyxin biosynthesis (PPYC2_03650-03865), one gene cluster related to tridecaptin synthesis (PPYC2_14680-14845), and one fusaricidin synthetic gene cluster (PPYC2_26180-26360). The complete genome sequence of *P. polymyxa* YC0573 contributes to the study of the molecular mechanisms of this species for plant growth promotion and of the benefits of its application for biological control.

### Accession number(s).

The complete genome sequence of *P. polymyxa* YC0573 has been deposited in GenBank under the accession number CP017968. The version described in this paper is the first version.
